# Empagliflozin Reduced Mortality and Hospitalization for Heart Failure Across the Spectrum of Cardiovascular Risk in the EMPA-REG OUTCOME Trial

**DOI:** 10.1161/CIRCULATIONAHA.118.037778

**Published:** 2018-12-06

**Authors:** David Fitchett, Silvio E. Inzucchi, Christopher P. Cannon, Darren K. McGuire, Benjamin M. Scirica, Odd Erik Johansen, Steven Sambevski, Stefan Kaspers, Egon Pfarr, Jyothis T. George, Bernard Zinman

**Affiliations:** 1 St Michael’s Hospital, Division of Cardiology, University of Toronto, Canada (D.F.).; 2 Section of Endocrinology, Yale University School of Medicine, New Haven, CT (S.E.I.).; 3 Cardiovascular Division, Brigham and Women’s Hospital, and Harvard Medical School, Boston, MA (C.P.C., B.M.S.).; 4 University of Texas Southwestern Medical Center, Dallas (D.K.M.).; 5 Boehringer Ingelheim Norway KS, Asker (O.E.J.).; 6 Boehringer Ingelheim International GmbH, Ingelheim, Germany (S.S., S.K., E.P., J.T.G.).; 7 Lunenfeld-Tanenbaum Research Institute, Mount Sinai Hospital, University of Toronto, Canada (B.Z.).

**Keywords:** cardiovascular diseases, carotid artery diseases, death, sudden, cardiac, diabetes mellitus, type 2, sodium-glucose transporter 2

## Abstract

**Background:**

In the EMPA-REG OUTCOME trial (BI 10773 [Empagliflozin] Cardiovascular Outcome Event Trial in Type 2 Diabetes Mellitus Patients) in patients with type 2 diabetes mellitus and atherosclerotic cardiovascular disease, in comparison with placebo, empagliflozin reduced the risks of 3-point major adverse cardiovascular events (3-point MACE), cardiovascular and all-cause death, and hospitalization for heart failure. We investigated whether these effects varied across the spectrum of baseline cardiovascular risk.

**Methods:**

Cardiovascular death, all-cause mortality, 3-point MACE, and hospitalization for heart failure in the pooled empagliflozin and placebo groups were analyzed in subgroups by prior myocardial infarction and stroke at baseline, and by estimated baseline cardiovascular risk based on the 10-point TIMI (Thrombolysis In Myocardial Infarction) Risk Score for Secondary Prevention.

**Results:**

Of 7020 patients who received the study drug, 65% had a prior myocardial infarction or stroke, and 12%, 40%, 30%, and 18% were at low, intermediate, high, and highest estimated cardiovascular risk according to TIMI Risk Score for Secondary Prevention (≤2, 3, 4, and ≥5 points, respectively). In the placebo group, 3-point MACE occurred during the trial in 7.3%, 9.4%, 12.6%, and 20.6% of patients at low, intermediate, high, and highest estimated baseline risk, respectively. Relative reductions in risk of cardiovascular death, all-cause mortality, 3-point MACE and hospitalization for heart failure with empagliflozin versus placebo were consistent in patients with and without prior myocardial infarction and/or stroke and across subgroups by TIMI Risk Score for Secondary Prevention at baseline (*P*>0.05 for randomized group-by-subgroup interactions).

**Conclusions:**

Despite all patients having atherosclerotic cardiovascular disease, patients in EMPA-REG OUTCOME demonstrated a broad risk spectrum for cardiovascular events. Reductions in key cardiovascular outcomes and mortality with empagliflozin versus placebo were consistent across the range of cardiovascular risk.

**Clinical Trial Registration:**

URL: https://www.clinicaltrials.gov. Unique identifier: NCT01131676.

## Abstract 2

Supplemental Digital Content is available in the text.

**Clinical Perspective**

**What Is New?**

- Among patients with type 2 diabetes mellitus and prevalent atherosclerotic cardiovascular disease, there is a 10-fold variation in future cardiovascular (CV) risk.
- In this group, individuals with or without a prior atherothrombotic event, and across the spectrum of predicted risk derived from baseline characteristics, have a similar reduction in CV mortality and hospitalization for heart failure with the sodium glucose cotransporter 2 inhibitor, empagliflozin.

**What Are the Clinical Implications?**

- The reduction of CV events with empagliflozin, in patients with type 2 diabetes mellitus and known atherosclerotic CV disease extends across the spectrum of underlying CV risk, and is not confined to patients at the highest risk.
- Empagliflozin should be considered in patients with type 2 diabetes mellitus and all forms of atherosclerotic CV disease to reduce CV mortality and hospitalization for heart failure.

Cardiovascular disease presents as a range of phenotypes that starts with the presence of multiple risk factors and progresses, largely because of atherothrombotic events, to myocardial infarction (MI), heart failure (HF), and death.^1,2^ However, myocardial dysfunction, HF, and cardiovascular death can occur independently of atherothrombosis, especially in patients with diabetes mellitus.^3,4^ Patients with type 2 diabetes mellitus (T2DM) are considered to be at very high risk for future adverse cardiovascular events.^5,6^ However, recent data show a broad spectrum of cardiovascular risk exists even in patients with cardiovascular disease and diabetes mellitus.^7^

Several stratification methods have been developed to predict the risk of primary cardiovascular events in individuals with cardiovascular disease risk factors^8,9^ and the risk of recurrent cardiovascular events in individuals with recent acute coronary syndrome.^10–12^ The Thrombolysis In Myocardial Infarction (TIMI) Risk Score for Secondary Prevention (TRS 2°P) is a risk stratification tool developed to predict recurrent cardiovascular events in patients with a history of MI^13^ and validated in other populations, including patients with stable atherosclerotic cardiovascular disease (ASCVD).^14,15^ The original version conferred 1 point for each of 9 cardiovascular risk factors: HF, hypertension, age ≥75 years, diabetes mellitus, prior stroke, prior coronary artery bypass graft surgery, peripheral vascular disease, estimated glomerular filtration rate <60 mL·min^1^·1.73 m^2^, and current smoking.^13^ To stratify risk in patients with stable ASCVD, the score was adapted to include prior MI as a risk factor to make a maximum 10-point score.^15^ The 10-point TRS 2°P was evaluated in patients with T2DM and high cardiovascular risk with data from the SAVOR TIMI 53 trial (Saxagliptin Assessment of Vascular Outcomes Recorded in Patients with Diabetes Mellitus-Thrombolysis in Myocardial Infarction 53)^7^ and validated for the prediction of major adverse cardiovascular events (MACE) using the cohort of patients with T2DM and high cardiovascular risk from the REACH observational registry (Reduction of Atherothrombosis for Continued Health).^7,16^

In the EMPA-REG OUTCOME trial (BI 10773 [Empagliflozin] Cardiovascular Outcome Event Trial in Type 2 Diabetes Mellitus Patients) in patients with T2DM and ASCVD, the sodium glucose cotransporter 2 inhibitor empagliflozin given in addition to standard of care reduced the risk of 3-point MACE (composite of cardiovascular death, nonfatal MI, or nonfatal stroke) by 14%, cardiovascular death by 38%, all-cause death by 32%, and hospitalization for heart failure (HHF) by 35% in comparison with placebo.^17^ ASCVD was defined as a prior atherothrombotic event (MI or stroke) and other vascular manifestations of ASCVD (multivessel coronary artery disease; single-vessel coronary artery disease with ischemia and unstable angina ≤12 months before consent or occlusive peripheral artery disease).^17^

Currently, it is uncertain whether patients with diabetes mellitus with other ASCVD risk factors yet no prior proven ASCVD derive cardiovascular benefits from sodium glucose cotransporter 2 inhibition. In the CANVAS trials program (Canagliflozin Cardiovascular Assessment Study), the subgroup with no ASCVD had no apparent reduction in risk for the primary outcome in comparison with patients with prevalent ASCVD at trial entry, with a *P* value for interaction of 0.18. Consequently, it is important to determine whether patients at lower risk of cardiovascular events have a reduction of risk for cardiovascular outcomes with empagliflozin.

In the present analyses, we (1) assessed the spectrum of predicted and observed cardiovascular risk among patients with T2DM and prevalent ASCVD enrolled in the EMPA REG OUTCOME trial, and (2) investigated whether the effects of empagliflozin on cardiovascular outcomes and mortality varied across the spectrum of cardiovascular risk at baseline. We assessed the risk of these outcomes in subgroups of patients (1) with and without a prior atherothrombotic event (MI or stroke) at baseline, and (2) stratified by baseline cardiovascular risk estimated using the 10-point TRS 2°P.

## Methods

### Data Sharing

To ensure independent interpretation of clinical study results, Boehringer Ingelheim grants all external authors access to all relevant material, including participant-level clinical study data, and relevant material as needed by them to fulfill their role and obligations as authors under the International Committee of Medical Journal Editors criteria. Furthermore, clinical study documents (eg, study report, study protocol, statistical analysis plan) and participant clinical study data are available to be shared after publication of the primary manuscript in a peer-reviewed journal and if regulatory activities are complete and other criteria met per the BI Policy on Transparency and Publication of Clinical Study Data: https://trials.boehringer-ingelheim.com/transparency_policy.html.

Before providing access, documents will be examined, and, if necessary, redacted and the data will be deidentified, to protect the personal data of study participants and personnel, and to respect the boundaries of the informed consent of the study participants. Clinical Study Reports and Related Clinical Documents can be requested via this link:

https://trials.boehringer-ingelheim.com/trial_results/clinical_submission_documents.html.

All such requests will be governed by a Document Sharing Agreement. Bona fide, qualified scientific and medical researchers may request access to deidentified, analyzable participant clinical study data with corresponding documentation describing the structure and content of the data sets. On approval, and governed by a Data Sharing Agreement, data are shared in a secured data-access system for a limited period of 1 year, which may be extended on request. Researchers should use https://clinicalstudydatarequest.com to request access to study data.

### Trial Design

Patients with T2DM and hemoglobin A1c (HbA1c) 7% to 10%, established ASCVD, and estimated glomerular filtration rate ≥30 mL·min^1^·1.73m^2^ were randomly assigned to receive empagliflozin 10 mg, empagliflozin 25 mg, or placebo once daily in addition to standard of care. Investigators were encouraged to treat cardiovascular risk factors to achieve optimal standard of care according to local guidelines. The trial was to continue until ≥691 patients experienced an adjudicated primary outcome event (3-point MACE). Patients who prematurely discontinued study medication were followed for ascertainment of cardiovascular outcomes and vital status.^17^

The trial was conducted in accordance with the principles of the Declaration of Helsinki and the International Conference on Harmonization Good Clinical Practice guidelines and was approved by local authorities. An independent ethics committee or institutional review board approved the clinical protocol for each participating center. Patients provided written informed consent before entering the trial.

### Outcomes

We analyzed the risks of cardiovascular death, all-cause mortality, 3-point MACE, HHF, and the composite of HHF or cardiovascular death, and the effects of empagliflozin versus placebo on these outcomes in subgroups stratified by the presence or absence of prior MI or stroke. Furthermore, we analyzed the same risks and effects of empagliflozin in subgroups stratified based on 10-point TRS 2°P at baseline. Baseline HF used in the TRS 2°P was based on the Medical Dictionary for Regulatory Activities preferred term “cardiac failure congestive”. Patients were categorized into 4 groups based on 10-point TRS 2°P risk scores of ≤2 points, 3 points, 4 points, and ≥5 points. We labeled these low risk, intermediate risk, high risk, and highest risk, respectively. The subgroup analysis by the TRS 2°P risk score was not prespecified in the original statistical analysis.

### Analyses

Analyses were performed in patients treated with ≥1 dose of study drug. Differences in risk for the 2 doses of empagliflozin pooled versus placebo were compared by using multivariable Cox proportional hazards regression analyses including factors for age, sex, baseline body mass index, baseline estimated glomerular filtration rate, region, baseline HbA1c, and randomized group. Subgroup analyses by history of MI or stroke at baseline were performed using the main analysis model,^17^ with additional factors for prior MI or stroke and the interaction term of randomized group-by-prior MI or stroke. Subgroup analyses by TRS 2°P at baseline were performed using the main analysis model with additional factors for TRS 2°P category and the interaction term of randomized group-by-TRS 2°P category. *P* values for randomized group-by-subgroup interactions were obtained from tests of heterogeneity of treatment group differences among subgroups with no adjustment for multiple testing.

## Results

### Patients With and Without Prior MI or Stroke

Of 7020 patients who received study drug, 65% in both the empagliflozin pooled and placebo groups had a prior MI or stroke, whereas 35% had coronary artery disease and peripheral artery disease without a prior MI and stroke. Those with versus without a prior MI and stroke were more likely to be white; have HF; be taking β-blockers, mineralocorticoid receptor antagonists, statins, or clopidogrel; and less likely to have a diagnosis of T2DM for >10 years. Baseline characteristics were similar between the empagliflozin and placebo groups in both strata defined by prior MI or stroke (Table 1).

**Table 1.**
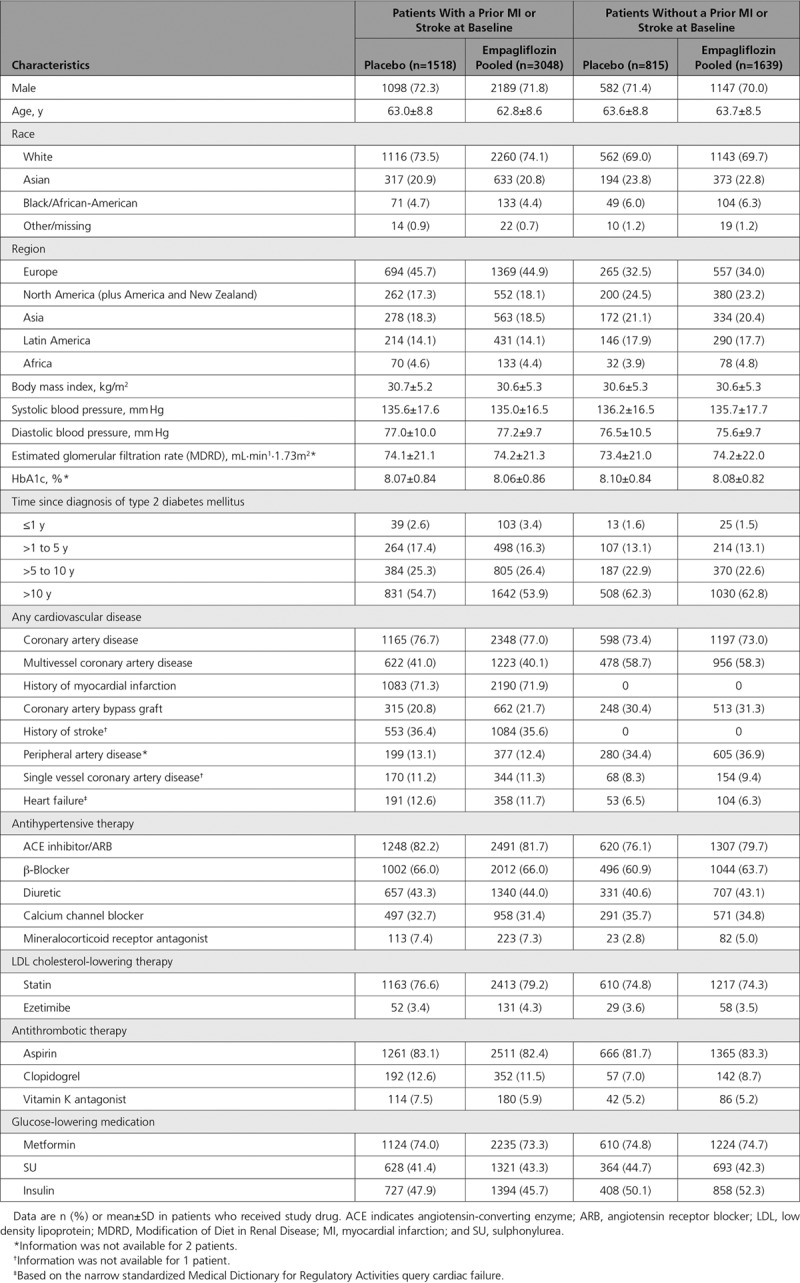
Baseline Characteristics in Patients With and Without a History of Myocardial Infarction or Stroke at Baseline

**Figure 1.**
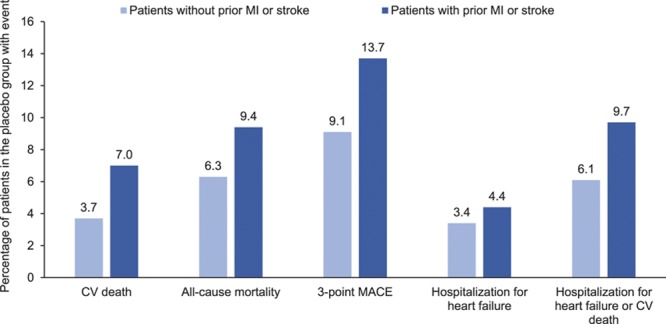
**Cardiovascular (CV) outcomes and mortality in the placebo group by presence or absence of prior myocardial infarction (MI) or stroke at baseline.** Descriptive data in patients who received study drug. MACE indicates major adverse cardiovascular events.

**Figure 2.**
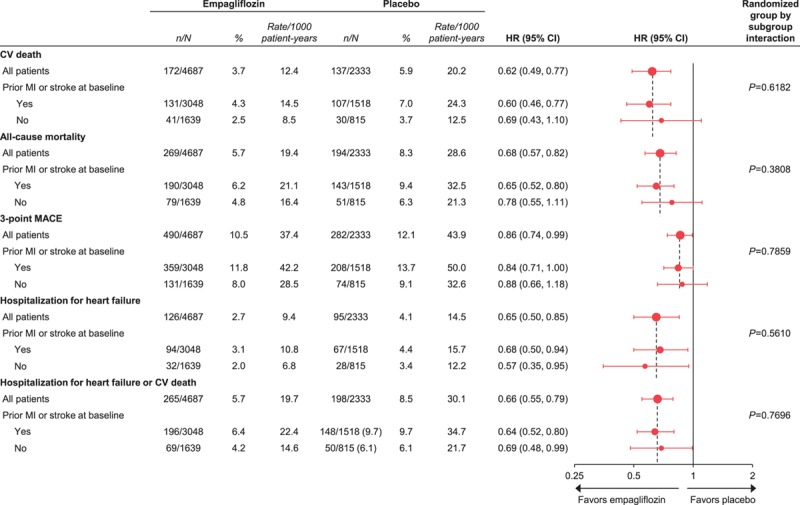
**Cardiovascular (CV) outcomes and mortality with empagliflozin versus placebo in subgroups stratified by presence or absence of prior myocardial infarction (MI) and stroke at baseline.** Cox proportional hazards regression analyses in patients who received study drug. HR indicates hazard ratio; and MACE, major adverse cardiovascular events.

**Figure 3.**
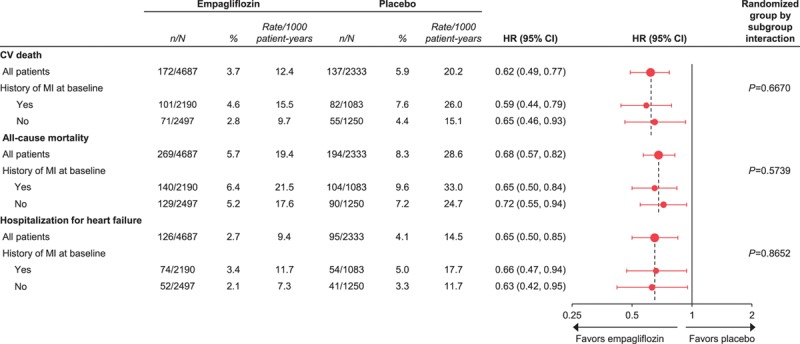
**Cardiovascular (CV) outcomes and mortality with empagliflozin versus placebo by history of myocardial infarction (MI) at baseline.** Cox regression analyses in patients who received study drug. HR indicates hazard ratio.

**Figure 4.**
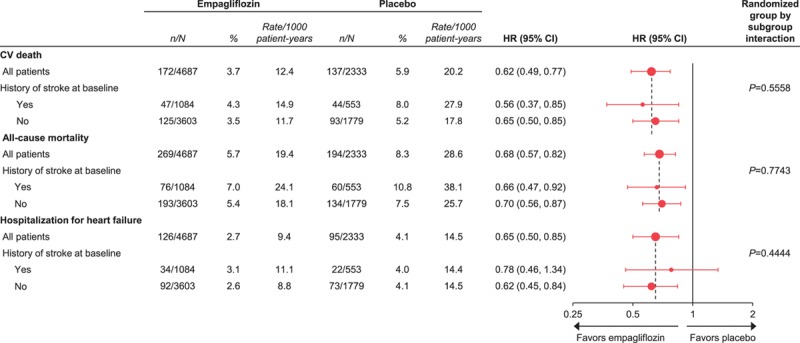
**Cardiovascular (CV) outcomes and mortality with empagliflozin versus placebo by history of stroke at baseline.** Cox regression analyses in patients who received study drug.

In the placebo group, cardiovascular death occurred in 7.0% of patients with a prior MI or stroke and 3.7% of patients without (Figure 1). The proportions of patients with all-cause mortality, 3-point MACE, and HF outcomes were also greater in those with versus without a prior MI or stroke (Figure 1). The reductions in risk of cardiovascular death, all-cause mortality, 3-point MACE, HHF, and the composite of HHF or cardiovascular death with empagliflozin were consistent in patients with and without a prior MI or stroke (*P*>0.05 for treatment by subgroup interactions) (Figure 2). The effects of empagliflozin versus placebo on cardiovascular death, all-cause mortality, and HHF were also consistent in subgroups by prior MI and by prior stroke (*P*>0.05 for randomized group-by-subgroup interaction tests) (Figures 3 and 4).

### Patients Stratified by TRS 2°P at Baseline

Based on the TRS 2°P at baseline, 12%, 40%, 30%, and 18% of patients, respectively, were at low, intermediate, high, and highest estimated cardiovascular risk. These proportions were similar between the empagliflozin and placebo groups: low cardiovascular risk, 12.0% versus 11.8% with empagliflozin versus placebo; intermediate cardiovascular risk, 39.9% versus 41.2%; high cardiovascular risk, 30.9% versus 28.6%; and highest cardiovascular risk, 17.2% versus 18.4%. The distribution of patients across all risk score categories is shown in Figure I in the online-only Data Supplement. Baseline characteristics were similar between the empagliflozin and placebo groups across the 4 estimated cardiovascular risk categories (Table 2).

**Table 2.**
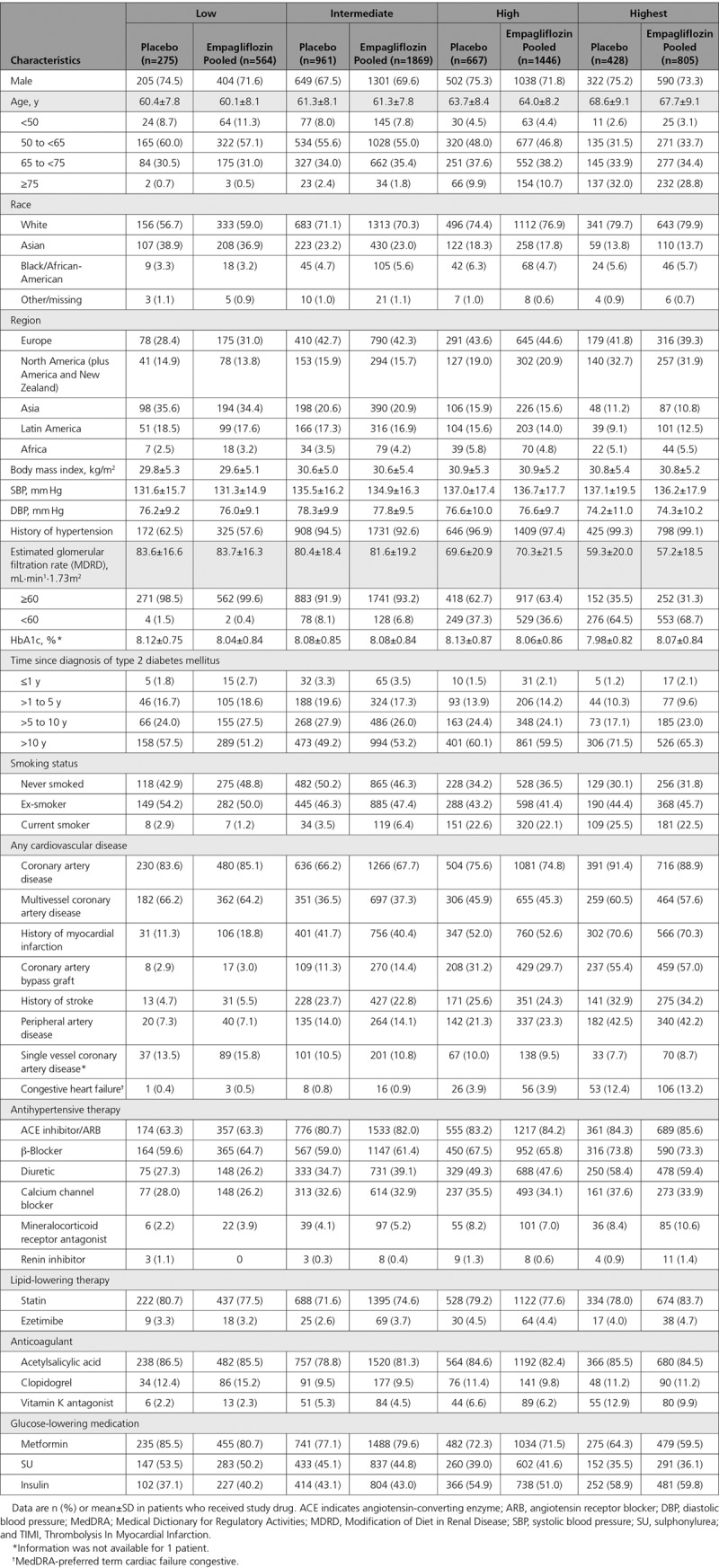
Baseline Characteristics by Estimated Cardiovascular Risk at Baseline According to the 10-point TIMI Risk Score for Secondary Prevention: Low, ≤2 points; Intermediate, 3 points; High, 4 points; Highest, ≥5 points.

**Figure 5.**
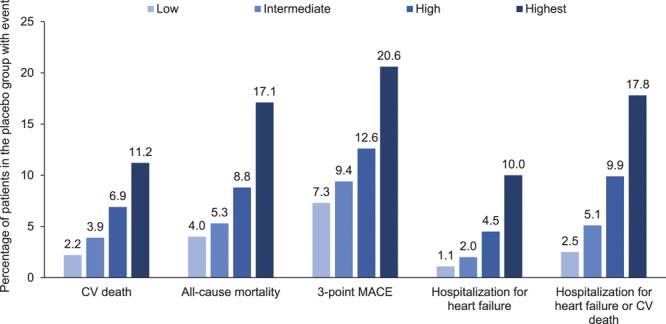
**Cardiovascular (CV) outcomes and mortality in the placebo group by estimated CV risk according to 10-point TIMI Risk Score for Secondary Prevention at baseline: low, ≤2 points; intermediate, 3 points; high, 4 points; and highest, ≥5 points.** Descriptive data in patients who received study drug. MACE indicates major adverse cardiovascular events.

**Figure 6.**
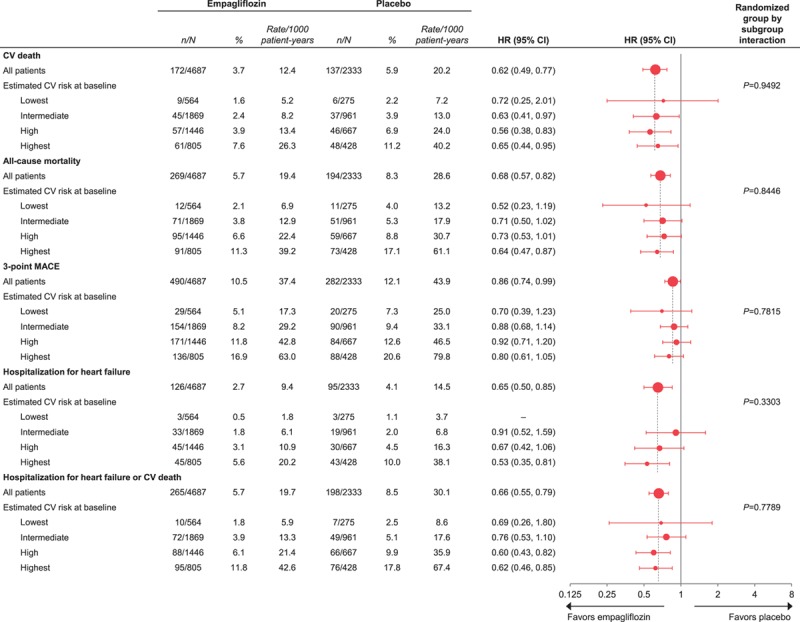
**Cardiovascular (CV) outcomes and mortality with empagliflozin versus placebo by estimated CV risk according to 10-point TIMI Risk Score for Secondary Prevention at baseline: low, ≤2 points; intermediate, 3 points; high, 4 points; and highest, ≥5 points.** Cox proportional hazards regression analyses in patients who received study drug. HR indicates hazard ratio; and MACE, major adverse cardiovascular events.

In the placebo group, the proportion of patients with cardiovascular death ranged from 2.2% to 11.2%, and the proportion with HHF ranged from 1.1% to 10.0%, across the subgroups by TRS 2°P at baseline. The proportions of patients with all-cause mortality and 3-point MACE also increased with greater TRS 2°P at baseline (Figure 5). The reductions in risk of cardiovascular death, all-cause mortality, 3-point MACE, HHF, and HHF or cardiovascular death with empagliflozin versus placebo were consistent across subgroups by baseline TRS 2°P (*P*>0.05 for randomized group-by-subgroup interaction tests) (Figure 6).

## Discussion

Patients with T2DM and established ASCVD are considered to be at very high risk of cardiovascular events such as cardiovascular death, myocardial infarction, stroke, or HF. In general, the greater the burden of risk factors and cardiovascular disease in patients with T2DM, the higher the risk for subsequent cardiovascular events.^7^ Patients with a prior MI or stroke, especially those with T2DM, are at much higher cardiovascular risk than those without such a history^18–20^ and carry a substantial public health burden.^21^ Even if risk factor targets are met with management of glycemia, blood pressure, low-density lipoprotein cholesterol, smoking cessation, physical activity, and use of antithrombotic agents, patients with T2DM and established ASCVD remain at high risk of cardiovascular events.^22,23^

In the EMPA REG OUTCOME trial, all patients had T2DM and established ASCVD. However, results from the present analyses show that, in this population that is usually considered to have a very high cardiovascular risk for future events, there is a wide spectrum of risk. Patients with a history of an atherothrombotic event (MI or stroke) had 2-fold greater risk of cardiovascular death than individuals with ASCVD but no prior MI or stroke. The TRS 2°P identified a range of estimated cardiovascular risk, with observed rates of cardiovascular mortality >5-fold higher in the very-high-risk group (40.2 events per 1000 patient-years) in comparison with the lower risk cohort (7.2 events per 1000 patient-years). All-cause mortality, 3-point MACE, HHF, or the combined outcome of HHF or cardiovascular death were 4- to 10-fold higher in the very-high-risk subgroup than in the lower-risk patients. A similarly wide spectrum of cardiovascular risk was observed in the SAVOR TIMI 53 trial in patients with established ASCVD or at high ASCVD risk, with the 2-year incidence of 3-point MACE outcomes ranging from 0.9% to 19.8% across subgroups defined by TRS 2°P.^7^

We investigated whether the effects of empagliflozin on key cardiovascular outcomes and mortality in the EMPA-REG OUTCOME trial varied by cardiovascular risk estimated at baseline. Reductions in risk for cardiovascular outcomes and mortality with empagliflozin versus placebo were observed across the spectrum of baseline cardiovascular risk, without evidence of heterogeneity of treatment effects in patients with or without a prior MI or stroke or across the spectrum of baseline estimated risk by the TRS 2°P risk score. Empagliflozin reduced cardiovascular mortality, all-cause mortality, and HHF irrespective of estimated cardiovascular risk based on baseline TRS 2°P, even in patients with a cardiovascular risk level comparable to that observed in patients with no established CVD.^24^

These observations are consistent with other analyses of the EMPA-REG OUTCOME trial that showed a benefit from empagliflozin across a wide spectrum of baseline conditions and risk. Consistent effects of empagliflozin in reducing cardiovascular outcomes and mortality have also been demonstrated in subgroups with and without a history of coronary artery bypass graft surgery,^25^ and with or without peripheral artery disease at baseline.^26^ In addition, the reduction in risk of HHF with empagliflozin has been shown to be consistent in patients with and without HF at baseline^27^ and across the spectrum of HF risk assessed using the 9-variable Health ABC risk score based on age, coronary artery disease, systolic blood pressure, heart rate, left ventricular hypertrophy, smoking, serum albumin, fasting blood glucose, and creatinine.^28,29^ Consistent effects of empagliflozin on cardiovascular outcomes have also been demonstrated across subgroups based on age,^30,31^ sex,^32^ HbA1c,^17,33,34^ kidney function,^35^ hypertension and use of antihypertensive therapy,^17^ use of statins or ezetimibe,^17^ and smoking status,^36^ and in analyses adjusting for control of HbA1c, blood pressure, and low-density lipoprotein cholesterol during the trial.^37,38^ Thus, the cardiovascular benefits of empagliflozin appear to apply irrespective of the degree of cardiovascular risk and cardiovascular risk reduction strategies in patients who have T2DM with prevalent ASCVD.

The mechanisms responsible for the reductions in cardiovascular risk with empagliflozin remain to be fully elucidated. By blocking the action of sodium glucose cotransporter 2 in the proximal tubule, empagliflozin reduces renal glucose reabsorption and increases urinary glucose excretion. This is accompanied by transient natriuresis and increases in urine volume.^39,40^ Retaining glucose, sodium, and water in the proximal tubule leads to secondary downstream effects on glucose metabolism and salt and water handling. This is associated with reductions in plasma volume,^41^ increases in hematocrit and hemoglobin,^35,42^ and reductions in arterial stiffness and vascular resistance.^43^ In an exploratory mediation analysis, changes in markers of plasma volume appeared to be important mediators of the reduction in risk of cardiovascular death with empagliflozin.^42^ Other proposed mechanisms include the fuel hypothesis, with ketone bodies (which circulate in increased concentrations after sodium glucose cotransporter 2 inhibition) being a more efficient metabolite for a stressed myocardium.^44^ Multiple analyses suggest that the protective cardiorenal effects of empagliflozin are largely independent of its glucose-lowering effects.^34,42,45^

Strengths of the present analyses include the large sample size, near-complete ascertainment of vital status (>99% of the trial population), and the prospective capture and central adjudication of cardiovascular events. Limitations include their post hoc nature and the lack of adjustment for in-trial changes in background medications.

In conclusion, patients with T2DM and prevalent ASCVD risk exhibit a wide variation in risk for subsequent cardiovascular outcomes, with risk stratified by prior MI/stroke and well stratified by the TRS 2°P. Empagliflozin has a robust treatment effect in reducing mortality and HHF across a spectrum of cardiovascular risk. These findings suggest that treatment with empagliflozin might benefit patients with T2DM and ASCVD irrespective of a history of MI or stroke and across the spectrum of estimated cardiovascular risk. The DECLARE trial (Dapagliflozin Effect on Cardiovascular Events), which included a large proportion of patients with no prior established ASCVD, has reported a statistically significant reduction in the coprimary combined end point of cardiovascular mortality or HHF, but no reduction of cardiovascular mortality.^46^

## Acknowledgments

Medical writing assistance, supported financially by Boehringer Ingelheim, was provided by E. Ng and W. Morris of FleishmanHillard Fishburn, London, UK, during the preparation of this article. The authors were fully responsible for all content and editorial decisions and were involved at all stages of manuscript development and have approved the final version.

## Sources of Funding

This work was supported by the Boehringer Ingelheim and Eli Lilly and Company Diabetes Alliance. Boehringer Ingelheim was involved in the design and conduct of the study; collection, analysis, and interpretation of data; and preparation of this manuscript.

## Disclosures

Dr Fitchett has received honoraria from Sanofi, Merck & Co., Amgen, AstraZeneca, Eli Lilly and Company and Boehringer Ingelheim. Dr Inzucchi has consulted for Janssen, vTv Therapeutics and Alere, served on Clinical Trial Steering/Executive Committees for Boehringer Ingelheim, AstraZeneca, Novo Nordisk, Sanofi/Lexicon Pharmaceuticals, Daiichi-Sankyo and Eisai (Thrombolysis in Myocardial Infarction [TIMI]) and served on Data Monitoring Committees for Intarcia Therapeutics, Inc. Dr Cannon has received research grants from Amgen, Boehringer Ingelheim, Bristol-Myers Squibb, Daiichi Sankyo, Janssen and Merck, and received consulting fees from Alnylam, Amarin, Amgen, Boehringer Ingelheim, Bristol-Myers Squibb, Eisai, Janssen, Kowa, Merck, Pfizer, Regeneron and Sanofi. Dr McGuire has received honoraria for clinical trials leadership from AstraZeneca, Sanofi Aventis, Janssen, Boehringer Ingelheim, Merck & Co, Novo Nordisk, Lexicon, Eisai, GlaxoSmithKline and Esperion, and honoraria for consultancy from AstraZeneca, Sanofi Aventis, Lilly US, Boehringer Ingelheim, Merck & Co, Pfizer, Novo Nordisk and Metavant. Dr Scirica reports research grants via Brigham and Women’s Hospital from AstraZeneca, Eisai, Novartis and Merck, consulting fees from AstraZeneca, Biogen Idec, Boehringer Ingelheim, Covance, Dr. Reddy’s Laboratory, Eisai, Elsevier Practice Update Cardiology, GlaxoSmithKline, Lexicon, Merck, NovoNordisk, Sanofi and St. Jude’s Medical, and equity in Health[at]Scale. Drs Johansen, Sambevski, Kaspers, Pfarr, and George are employees of Boehringer Ingelheim. Dr Zinman has received research grants awarded to his institution from Boehringer Ingelheim, AstraZeneca and Novo Nordisk, and honoraria from Janssen, Sanofi, Eli Lilly and Company, Boehringer Ingelheim, Novo Nordisk and Merck.

## Supplementary Material
