# Supplementary material for: Empagliflozin Reduced Mortality and Hospitalization for Heart Failure Across the Spectrum of Cardiovascular Risk in the EMPA-REG OUTCOME Trial
Source: Circulation. 2018 Dec 6;139(11):1384–95. doi: 10.1161/CIRCULATIONAHA.118.037778 (PMC6416009; doi:10.1161/CIRCULATIONAHA.118.037778)

**Empagliflozin Reduced Mortality and Hospitalization for Heart Failure Across the Spectrum of Cardiovascular Risk in the EMPA-REG OUTCOME Trial (Fitchett D, et al).**

**SUPPLEMENTAL MATERIAL**

**Contents**

Figure I. Summary of patients by baseline cardiovascular (CV) risk score categories (all categories) based on TIMI risk score − treated set…………………………….…………….2

**Figure I. Summary of patients by baseline cardiovascular (CV) risk score categories (all categories) based on TIMI risk score − treated set.**

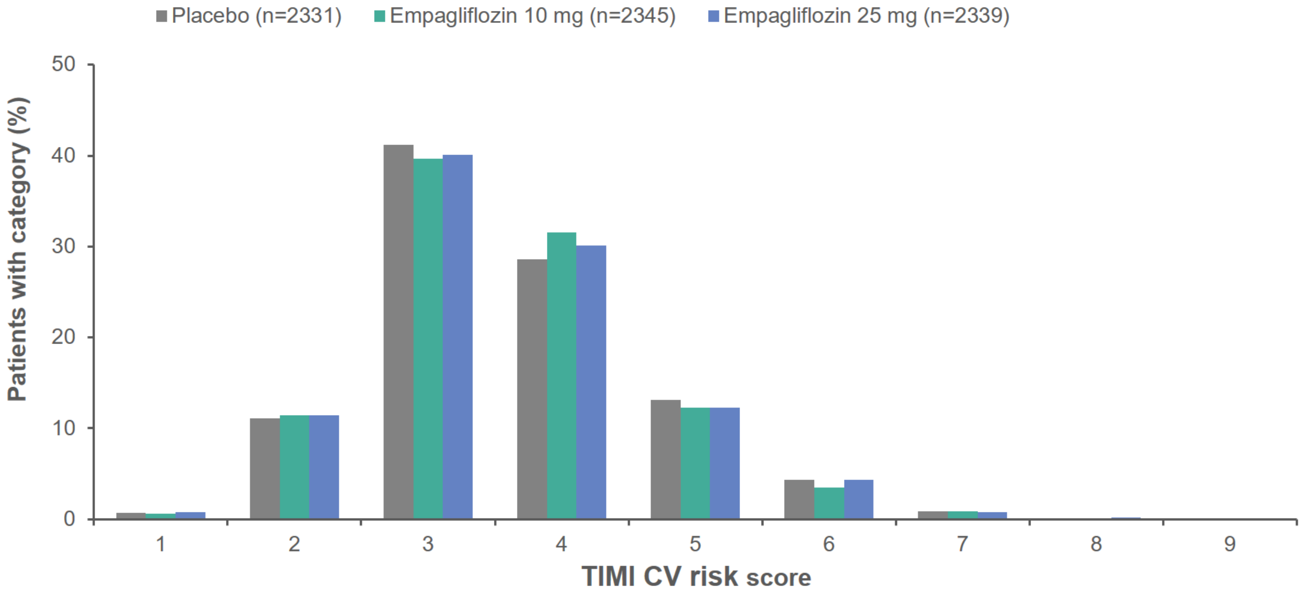

Supplement: Supplementary file 1 [file cir-139-1384-s001.pdf]
